# Effects of *Cordyceps militaris* solid medium on the growth performance, immunity parameters and intestinal health of broilers

**DOI:** 10.5713/ab.24.0791

**Published:** 2025-04-04

**Authors:** Xiaoya Wang, Jingyan Zhang, Kang Zhang, Guowei Xu, Zhiting Guo, Xueyan Gu, Saad Ahmad, Liping Huang, Shuqi Liu, Jianxi Li, Lei Wang

**Affiliations:** 1 Traditional Chinese Veterinary Technology Innovation Center of Gansu Province, Key Laboratory of Veterinary Pharmaceutical Development of Ministry of Agriculture and Rural Affairs of China, Lanzhou Institute of Husbandry and Pharmaceutical Sciences of Chinese Academy of Agricultural Sciences, Lanzhou, China

**Keywords:** Broiler, *Cordyceps militaris* Solid Medium, Growth Performance, Immunity, Intestinal Health

## Abstract

**Objective:**

This study aimed to investigate the impact of *Cordyceps militaris* solid medium (CMM) on the growth performance, immunity parameters, intestinal health and gut microbiota of broilers.

**Methods:**

A total of 320 male broilers were randomly allocated into 4 groups, consisting of 8 replicates and 10 birds per replication. The broilers were fed diets comprising the Control group, 0.02% CMM group (Low CMM), 0.2% CMM group (Mid CMM), or 2% CMM group (High CMM). Samples were collected on 14d and 28d of the experiment. Serum was collected for the determination of biochemical indices and immunoglobulins, the intestinal morphology of the duodenum, jejunum and ileum was observed, and cecum contents were collected for 16S rRNA gene sequencing.

**Results:**

Over the course of the 28d trial, the broilers fed CMM had a higher average daily gain and lower feed conversion rate than the control broilers at Days 14 and 28. Notably, compared with those of the Control group, the Low CMM diet increased the thymus and bursa indices significantly (p<0.05). Enzyme-linked immunosorbent assay revealed that CMM increased the IgA, IgM, and IgG levels in the serum, and the IgA level in the Low and Mid CMM groups was significantly higher than that in the Control group (p<0.05). A Low CMM diet showed the strongest ability to increase villus height (VH) and villus height/crypt depth, and CMM affected the relative mRNA expression levels of inflammatory factors in jejunal tissue. The 16S rRNA results showed that the CMM group presented a greater relative abundance of *Firmicutes* and a higher *Firmicutes/Bacteroidetes* ratio than did the Control group at 14 d and 28 d, and the *Deferribacteres* abundance also increased (14 d). At the genus level, a Low CMM diet decreased Bacteroides abundance (p<0.05) but increased the abundance of *Synergistetes* (p>0.05).

**Conclusion:**

These results illustrate that 0.02% CMM in the diet can promote growth performance, increase immunity, improve intestinal morphology, and regulate the intestinal flora of broilers.

## INTRODUCTION

Antibiotics have been extensively employed in poultry breeding to optimize growth performance and enhance health. However, misuse and/or overuse of antibiotics have resulted in increased emergence and dissemination of drug-resistant bacteria [1]. Furthermore, research has revealed that excessive antibiotic treatment may affect host energy metabolism and cause health problems [2]. The incorporation of antibiotics into animal feed has been subjected to regulatory restrictions in several countries, including China, the European Union and the United States [3,4]. This restriction poses a challenge to the development of the livestock and poultry industries, necessitating the exploration of alternative feed additives capable of enhancing the growth performance and immune capacity of livestock and poultry. Previous investigations have provided evidence that plant-based feed additives can improve growth performance, immunological function, and microbial regulation. Abdelatty et al [5] observed that dietary supplementation with Manjiang red leaf powder had favorable effects on gut morphology and function in broilers. Farahat et al [6] found that plant extracts altered the intestinal morphology and VH/CD ratio while increasing the *Lactobacillus* population. Therefore, plant-based feed additives are expected to become viable alternatives to antibiotics and are increasingly being studied. Intestinal health is a crucial determinant of chicken growth performance, and the intestinal tract is an essential immune organ that maintains body homeostasis via selective barrier functions. Therefore, ensuring intestinal health remains a top priority in animal farming [7]. The symbiotic equilibrium between the gut microbiota and intestines influences the health status, growth performance, and developmental processes of chickens [7]. However, variations in intestinal morphology and microbiota composition have been observed among broilers at different stages of development [8]. Consequently, it is vital to explore how feed additives impact chicken health and growth performance through changes in intestinal health.

*Cordyceps militaris* solid medium (CMM) is a byproduct derived from the artificial cultivation of *Cordyceps militaris*. The culture technology of *Cordyceps militaris* has developed rapidly, and artificial large-scale cultivation has been realized. A large amount of CMM remains after *Cordyceps militaris* is harvested, with more than 1 kg of CMM produced for every 1 kg of *Cordyceps militaris* harvested [9]. Most CMM is currently treated as waste, resulting in resource waste and environmental pollution. *Cordyceps militaris* is a fungus that can be used as both a medicine and food; it contains a variety of nutrients and active ingredients, such as cordycepin, cordycepic acid and cordyceps polysaccharide, and has a variety of biological activities [10]. CMM also contains valuable active components such as cordycepin and cordyceps acid [11]. Several reports have shown that CMM possesses biological activity and functional benefits. For example, Cheng et al. found that the addition of CMM to feed at a 0.5% ratio affected the production performance and egg quality of laying hens [12]. Moreover, Hsieh et al demonstrated that the addition of 1% CMM to broiler meals improved intestinal health and growth performance and also enhanced antioxidant capacity [13]. Moreover, Wang et al reported increased egg production and reduced cholesterol levels in laying hens supplemented with 2% CMM in feed [14]. Dai et al. found that adding CMM to the diet at 0.01% to 0.03% could improve the performance and carcass quality of finishing pigs [15]. These findings suggest that CMM as a feed additive exhibit promising efficacy, underscoring the importance of further research and development in this area. However, the addition of CMM as a feed additive varies significantly across studies, which hinders the application of CMM in livestock and poultry farming. It is also not clear whether CMM has a regulatory effect on chicken intestinal flora. Thus, in this study, three doses of CMM in feed (0.02%, 0.2%, and 2%) were used to systematically compare the effects of different doses of CMM on the growth performance, intestinal morphology, immune capacity and intestinal microbes of broilers to provide a basis for determining the effective dose of CMM as a feed additive.

## MATERIALS AND METHODS

The experimental procedure received approval from the Lanzhou Animal Husbandry and Veterinary Medicine Animal Ethics Committee and was performed in strict compliance with the guidelines for the management of experimental animals in Gansu Province (approval number: 2023-007).

### Animal experimental design

Before the test was carried out, the coop equipment and ground were comprehensively cleaned and disinfected, the drinking water line was soaked and disinfected for 24 h, and the water line was rinsed with running water. Broilers are kept in temperature-controlled sheds at 27±2°C. The light was combined natural light and artificial light, and the ventilation system included automatic ventilation with negative pressure machinery. The chicken coops were cleaned and disinfected every week, and the chicken manure was mechanically removed 1–3 times a week to maintain a hygienic environment. A total of 320 male broilers (28-day-old yellow-feathered) were selected for this study, and the experimental period lasted for 28 d. Broilers were randomly divided into 4 groups with 8 replicate cages per group and 10 broilers per cage (1.5×0.6× 0.72 m^3^/cage). The groups were assigned the following diets: base diet (Control), 0.02% CMM (Low CMM), 0.2% CMM (Mid CMM) and 2% CMM (High CMM). The composition and nutrient levels of the base diet at 1 to 28 d and 29 to 56 d are shown in Table 1, with reference to Chicken Feeding Standards (Nutrient requirements of yellow chickens: NY/T3645–2020) [16]. The CMM was obtained from Xuzhou Hongyu Agricultural Science and Technology Co., Ltd. (Jiangsu, China), and the composition of the CMM, including amino acids and peptides, phenylpropanoids and polyketides, alkaloids, carbohydrates, triterpenoids and fatty acids, was analyzed via UHPLC-MS/MS, as shown in the supplementary materials. The nutrient levels of CMM, including crude protein, coarse fiber, crude fat, crude ash, calcium, phosphorus, and hydration, are shown in Table 2. Body weight (BW) and average daily feeding intake (ADFI) were recorded on Days 1, 14, and 28, and the average daily gain (ADG) and feed conversion rate (FCR) of each group were calculated.

### Sample collection

At 14 and 28 d after CMM treatment, one broiler was randomly selected from each of the eight replicates from the four groups. A 12 h fasting period was implemented for all broilers in the experimental group before weighing. Blood was taken from the subwing veins using a blood collection needle (0.55 m×19 mm) and a vacuum blood collection vessel, and the serum was collected by centrifugation (1,000 g/min, 15 min). The bursa, spleen, and thymus were extracted immediately and weighed, after which the organ indices were calculated (organ index = organ weight/BW×100%). After intestinal tissues and cecum contents were collected, segments of the small intestine were cut to approximately 2 cm and subsequently sliced, with the remaining samples were preserved at −80°C.

### Immune factors and serum biochemical indicators

The levels of IgG, IgA, and IgM in the serum were measured by the enzyme-linked immunosorbent assay (ELISA; Beijing Solarbio Technology Co., Ltd., Beijing, China). Blood biochemical indices were analyzed with an automatic biochemical analyzer (Mairui, Shenzhen, China).

### Intestinal morphology

The jejunum, ileum and duodenum of chickens were fixed with 4% paraformaldehyde, and the sections were prepared and stained with hematoxylin–eosin. The villus height (VH) and crypt depth (CD) were observed and imaged using a microscope. For each section, 3 fields of view under an optical microscope were recorded. ImageJ software was used to measure six relatively complete VH and CD in each tissue section and calculate the VH/CD ratio.

### The mRNA expression of inflammatory factors

Total jejunum RNA was prepared with an RNA extraction kit. The mRNA expression levels of interleukin (IL)-2, IL-6, IL-10, IL-1β, tumor necrosis factor-α (TNF-α) and interferon-γ (IFN-γ) were tested. The experimental procedure was carried out following the instructions of the kit (Hunan Ecorry Biological Engineering Co., Ltd., Hunan, China), and the primers used are presented in Table 3.

### Microbiota community analysis

The cecum contents were promptly frozen in liquid nitrogen for DNA extraction using a fecal DNA extraction kit (Beijing Solarbio Technology Co., Ltd., Beijing, China). Primers 338F (5′-ACTCCTACGGGAGGCAGCA-3′) and 806R (5′-GGAC TACHVGGGTWTCTAAT-3′) were used to amplify the high-variation region (V3–V4) of the bacterial 16S rRNA gene, which was then purified. The polymerase chain reaction products were purified, quantified, and homogenized to form sequencing libraries. Qualified libraries were sequenced using Sequel II to obtain 250 bp/300 bp counterpart reads. The primer sequences were identified and removed using Lima (V.1.7.0). Clean reads were obtained, and chimeric sequences were removed. The qualified sequences with a similarity threshold exceeding 97% were assigned to an operation taxonomic unit (OTU). Alpha and beta diversity calculations were performed to evaluate the species complexity and sample diversity. Bacterial abundance and diversity comparisons were conducted using a one-way analysis of variance. Differentially abundant taxa were identified using linear discriminant analysis effect size (LEfSe) analysis. The data were analyzed on the BMK Cloud (www.biocloud.net) platform.

### Statistical analysis

The data were processed using SPSS 21.0 analysis software. One-way analysis of variance and Tukey multiple comparison test was performed. All results are expressed as the mean and SEM, with distinct lowercase letters denoting significant differences (p<0.05). Images were produced using GraphPad Prism 8 software. The gut microbiota was analyzed on the BMK Cloud (https://www.biocloud.net) platform.

## RESULTS

### Growth performance

No mortality was observed in any of the groups during the study period. The indicators of growth performance are displayed in Table 4. The BW of the broilers in all groups increased steadily throughout the feeding period, with the Low CMM and Mid CMM groups having significantly higher final BWs (p*<*0.05) compared with that of the Control group. Analysis of the ADG index indicated that the CMM groups exhibited significantly higher ADG compared with that of the control during Days 1 to 14 (p*<*0.05). Specifically, the Low CMM and Mid CMM groups showed significant increases in ADG from Days 15 to 28 and from Days 1 to 28 (p<0.05). Additionally, the ADFI of the Low CMM and Mid CMM groups significantly decreased from Days 15 to 28 (p<0.05), whereas the High CMM group had increased ADFI in all phases. The addition of CMM (1 to 14 d) did not significantly affect the FCR (p>0.05). However, the FCR significantly decreased from Days for the Low CMM and Mid CMM groups, whereas the High CMM group showed a nonsignificant increase in FCR (p>0.05). Overall, the addition of 0.02% and 0.2% CMM promoted growth performance, whereas the addition of 2% CMM did not promote growth performance.

### Immune organ indices

The effects of CMM on immune organs are shown in Table 5. At 14 d, CMM had no significant effect on the spleen index (p>0.05), with only the Low CMM group showing an increase. The thymus indices of broilers in the Low CMM and Mid CMM groups increased, with the Low CMM group showing a significant effect on the thymus index (p<0.05). Both the Low CMM and Mid CMM groups showed significant increases in the bursa index (p<0.05). At 28 d, compared with those of the control group, the CMM groups showed no significant change in the spleen index (p>0.05), but exhibited increases in the thymus and bursa indices. Specifically, Low CMM was associated with a significant increase in the thymus index (p<0.05). Notably, Low CMM showed the greatest increase in the immune organ index at both 14 and 28 d.

### Immunoglobulin levels

**Figure 1:**
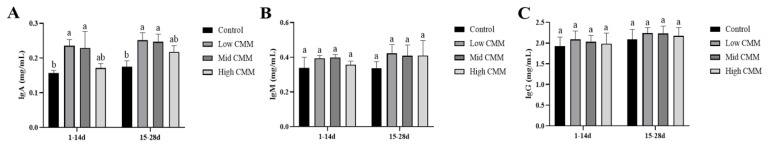
Effect of CMM on immunoglobulin levels. (A) IgA. (B) IgM. (C) IgG. ^a,b^ Different lowercase letters indicate significant differences, p<0.05, n = 6. CMM, *Cordyceps militaris* solid medium.

The IgA, IgM and IgG contents in the serum were quantified by ELISA. At 14 and 28 d, all doses of CMM did not significantly increase the contents of IgM and IgG (p>0.05). Low CMM and Mid CMM groups showed significantly increased IgA levels (p<0.05), but the High CMM group was associated with low IgA levels (Figure 1), which is consistent with the effects of High CMM doses on growth performance.

### Serum biochemical analysis

The serum biochemical results are presented in Table 6. On day 14 and 28, all indexes in all CMM groups showed no significant (p>0.05) alterations compared to the Control group (p>0.05). The levels of total protein (TP) and albumin (ALB) in the serum increased in the CMM groups (p>0.05), whereas the aspartate aminotransferase (AST), alanine aminotransferase (ALT), triglyceride (TG), total cholesterol (TCH), urea (UREA) and creatinine (CREA) levels decreased (p>0.05).

### Intestinal morphology

**Figure 2:**
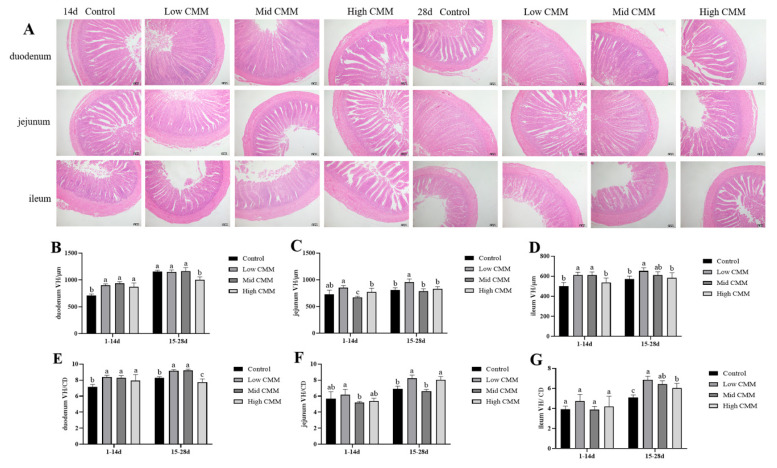
Intestinal morphology. (A) The structure of the duodenum, jejunum and ileum, 100× (B, C, and D, respectively). The VH of the duodenum, jejunum, and ileum (E, F, and G, respectively), and VH/CD of the duodenum, jejunum, and ileum. ^a–c^ Different lowercase letters indicate significant differences, p<0.05, n = 6. CMM, *Cordyceps militaris* solid medium; VH, villus height; CD, crypt depth.

The intestinal morphology and statistical analysis of the jejunum, ileum, and duodenum for all groups are shown in Figure 2A (14 d and 28 d). Notably, the intestinal villus and crypt structures in the CMM group were intact. Compared with the Control group, all the CMM doses increased the VH of the duodenum at 1 to 14 d (p<0.05), and the Low and Mid CMM increased the VH of the ileum at 1 to 14 d (p<0.05). Specifically, Low CMM significantly increased the ileum VH and duodenum VH/CD ratio (p<0.05), resulting in better performance at both 14 and 28 d.

### Relative mRNA expression of inflammatory factors in intestinal tissues

**Figure 3:**
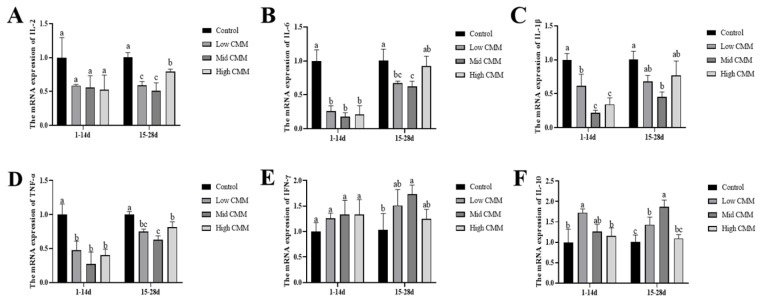
The effect of CMM on relative mRNA expression. (A) IL-2, (B) IL-6, (C) IL-1β, (D) TNF-α, (E) IFN-γ, and (F) IL-10. ^a–c^ Different lowercase letters indicate significant differences, p<0.05, n = 6. IL, interleukin; TNF, tumor necrosis factor; CMM, *Cordyceps militaris* solid medium; IFN, interferon-γ.

Figure 3 shows that the mRNA expression levels of IL-2, IL-6, IL-1β and TNF-α were downregulated, whereas the IL-10 and IFN-γ mRNA expression levels were upregulated at all doses of CMM at 14 and 28 d compared with those in the Control group. At 14 d, CMM significantly downregulated IL-6, IL-1β and TNF-α (p<0.05), whereas the decrease in IL-2 was not significant (p>0.05). Low CMM upregulated the expression of IL-10 mRNA (p<0.05). At 28 d, CMM significantly downregulated the expression of IL-2 and TNF-α mRNAs (p<0.05), whereas Mid CMM significantly reduced IL-1β and increased IFN-γ (p<0.05). Additionally, Low CMM and Mid CMM decreased the IL-6 level and significantly increased the IL-10 level (p<0.05).

### Gut microbiota

**Figure 4:**
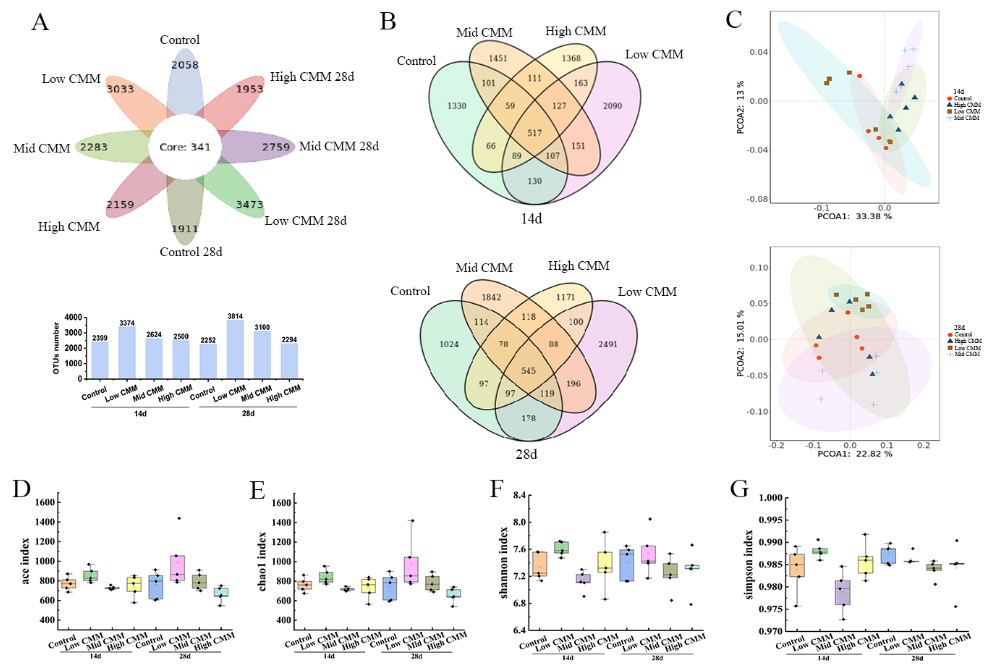
Microbiota analysis on OTU level. (A) Flower chart. (B) Venn diagram. (C) PCoA. (D) ACE. (E) Chao 1. (F) Shannon. (G) Simpson. n = 5. CMM, *Cordyceps militaris* solid medium; PCoA, Principal Coordinates Analysis; OTU, operation taxonomic unit; ACE, abundance-based Coverage Estimator.

The flora and number of OTUs in each group are presented in Figure 4A. On Day 14, the numbers of OTUs in the Control, Low CMM, Mid CMM and High CMM groups were 2,399, 3,374, 2,624, and 2,500, respectively. By Day 28, these OTU numbers were 2252, 3,814, 3,100, and, 2294, respectively, with the CMM groups consistently exhibiting higher OTU numbers than the Control group. To compare the OTUs among the four groups, a Venn diagram (Figure 4B) was generated, revealing that the number of unique OTUs in the CMM groups surpassed that in the Control group, with Low CMM having the highest number of unique OTUs at both time points. Specifically, the Low CMM treatment resulted in 975 (Day 14) and 1562 (Day 28) more unique OTUs than did the Control. Analysis of the Principal Coordinates Analysis diagram in Figure 4C revealed that the distance between samples from the Low CMM and Control groups was shortest on Day 14 (with the largest overlap area). By Day 28, the samples from all groups exhibited a scattered distribution, except for those from the Low CMM group, which showed a relatively concentrated distribution. Figure 4D and 4E show that the ACE and Chao1 indices of the Low CMM group surpassed those of the control group, indicating increased richness and diversity of species. Notably, the Mid CMM and High CMM groups did not exhibit improvements in these indices at 14 d; the Low CMM group demonstrated the most favorable performance even at 28 d. Furthermore, Low CMM improved the Shannon and Simpson indices at both Day 14 and Day 28 compared with those of the Control group, indicating increased species diversity. These results suggest that Low CMM supplementation increased species richness and diversity, resulting in the best performance at both time points.

#### Composition of phylum

**Figure 5:**
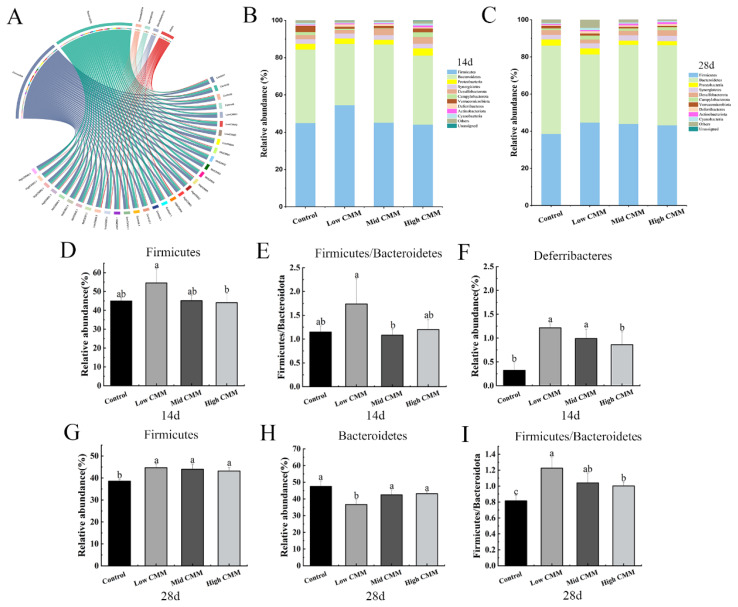
Composition analysis of microflora at the phylum level. (A) Circlize draft. (B) Top ten bacteria on phylum (day 14). (C) Top ten bacteria on phylum (day 28). (D) Relative abundance of *Firmicutes* at day 14. (E) *Firmicutes/Bacteroidetes* ratio at day 14. (F) Relative proportion of *Deferribacterota* day 14. (G) Relative proportion of Firmicutes day 28. (H) Relative proportion of Bacteroidetes day 28. (I) *Firmicutes/Bacteroidetes* ratio day 28. ^a–c^ Different lowercase letters indicate significant differences, p<0.05, n = 5. CMM, *Cordyceps militaris* solid medium.

Figure 5A shows the relationships between the samples and phylum, revealing that a total of 11 phylum was detected in the samples. The dominant phylum was similar among samples but present in different proportions, with *Firmicutes* and *Bacteroidetes* accounting for more than 80% of the phylum. The abundance of *Proteobacteria*, *Synergistetes* and *Desulfobacteres* were relatively low. Specifically, the proportion of *Firmicutes* in the Control, Low CMM, Mid CMM and High CMM groups were 44.95%, 54.55%, 45.16% and 44.11%, respectively. Figure 5D and 5E indicate that both Low CMM and Mid CMM increased the abundance of *Firmicutes*. Similarly, Low CMM increased the *Firmicutes*/*Bacteroidetes* ratio. The proportion of *Deferribacteres* in the CMM groups was higher than that in the Control group, with that in the Low CMM and Mid CMM groups increasing significantly (p<0.05) (Figure 5F). The composition and structure of the flora in each group at the phylum level on Day 28 is presented in Figure 5C. The proportions of *Firmicutes* were 38.59%, 44.69%, 44.00% and 43.19% in the Control group, Low CMM, Mid CMM and High CMM groups, respectively. The respective proportions of *Bacteroidetes* were 47.53%, 36.70%, 42.51% and 43.16%, and the ratios were 0.811, 1.217, 1.035, and 1.001, as shown in Figure 5G-I. Compared with that in the Control group, the proportion of *Firmicutes* in the CMM group increased significantly (p<0.05), whereas the proportion of *Bacteroidetes* decreased, leading to a significant increase in the *Firmicutes*/*Bacteroidetes* ratio (p<0.05).

#### Composition on genus

**Figure 6:**
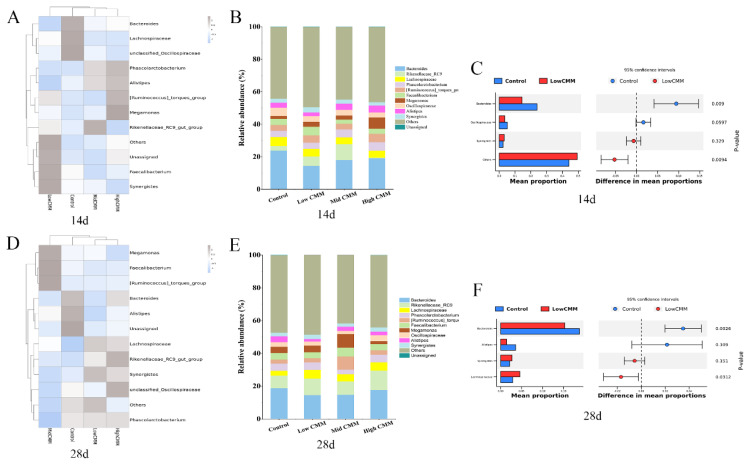
Composition analysis of microflora at the genus level. (A) Heatmap at 14 d. (B) Bar plot at 14 d. (C) Stamp analysis of the Control group and Low CMM group-14 d. (D) Heat map-28 d. (E) Barplot chart-28 d. (F) Stamp analysis between control group and low CMM group-28 d. CMM, *Cordyceps militaris* solid medium.

The classification of different genera in the samples revealed further taxonomic differences; 799 genera were identified. The characteristics of the 12 genera with the highest abundance were analyzed through a heatmap, in which *Bacteroides*, *Ruminococcus_torques_group* and *Alistipes* had the highest proportions of representative genera. At Day 14, *Bacteroides*, *Oscillospira* and *Lachnospira* were the most abundant bacteria in the Control group, whereas *Faecalibacterium* and *Synergistes* were the most abundant bacteria in the Low CMM group (Figures 6A, 6B). The proportions of *Alistipes* and *Ruminococcus*_*torques*_*group* were highest in the High CMM group. Figure 6C shows the difference analysis diagram (Stamp) between the Low CMM group and the control group at 14 d. The proportion of *Bacteroides* in the Low CMM group was significantly lower than that in the Control group (p<0.05), whereas the proportion of *Oscillospiraceae* was decreased and that of *Synergistes* was increased. On Day 28, *Bacteroides* and *Alistipes* accounted for the highest proportions in the Control group, and *Synergistes* was present in the highest proportion in the Low CMM group (Figures 6D, 6E). The results shown in Figure 6F indicate that the proportions of *Bacteroides* and *Alistipes* in the Low CMM treatment group were significantly lower than those in the Control group (p<0.05), whereas the proportions of *Synergistes* and *Lachnospiraceae* were greater than those in the Control group. At 14 d and 28 d, the Low CMM group exhibited an increase in the proportion of *Synergistes*, and the *Bacteroides* abundance was reduced significantly (p<0.05).

#### Linear discriminant analysis effect size analysis

The LEfSe analysis revealed the key microbial players in each group. The cladogram (7A) and linear discriminant analysis diagram (7B) show that at 14 d, g_*Lachnospiraceae*, p_*Firmicutes*, s_*Bacteroides*_*sp*_*Marsille_P3116* and g_*Alistipes* played the most important roles, in that order. On Day 28, f_*Muribaulaceae*, g_*Rikeneliaceae*_*RC9*_*gut-group*, o_*Lachnospirales* and o_*Bcteroidales* played the most important roles in the four groups, in that order.[Fig f7-ab-24-0791]

## DISCUSSION

In intensive livestock and poultry farming, antibiotics are commonly used to combat bacterial infections and diseases caused by pathogenic microorganisms. However, excessive and unnecessary use of prophylactic antibiotics is prevalent, leading to the rapid emergence of drug-resistant bacteria and environmental contamination [17]. Moreover, concerns about food safety have been raised due to the presence of drug residues. Several countries have implemented comprehensive prohibitions on the use of antibiotics in animal food products. Hence, alternative feed additives that substitute for antibiotics and are both safer and more environmentally friendly are needed.

In our study, we assessed the impact of different amounts of CMM supplement on the growth performance of broilers. The results indicated that the ADG of broilers supplemented with CMM was greater than that of the Control group and promoted the growth of broilers, but only at Low and Mid CMM concentrations (p<0.05). However, the ADFI and FCR in the High CMM group were significantly greater than those in the Control group. These findings indicate that the effect of CMM on the growth performance of broilers is not positively correlated with dose in the range from 0.02% to 2%. Cheng et al. also proposed that the effect of 0.5% CMM in feed on production performance and egg quality of laying hens was better than that of 1% CMM, indicating that higher doses of CMM do not result in the best performance [12]. In contrast, Wang reported that 2% CMM had a better effect on egg production in laying hens than did 1% CMM. The beneficial effect of CMM on growth performance may be due to the active ingredients contained in CMM. The active ingredient content should be considered when choosing the dosage of CMM as a feed additive, to achieve more effective production.

The developmental status of immune organs, including the thymus, spleen, and bursa, can serve as a direct reflection of immune competence in chickens and is a crucial indicator for assessing their growth status [18]. Our results showed that a Low CMM concentration significantly increased the bursa and thymus indices at 14 and 28 d, whereas a Mid CMM concentration significantly increased the bursa index at 14 d but did not alter other indices compared with those of the Control group (p>0.05). Notably, the spleen index of the High CMM group decreased (p>0.05), probably due to excessive dosage, which is consistent with the result that High CMM does not increase growth performance. These results highlight the importance of an appropriate CMM dosage in promoting the development of immune organs and initiating immune responses. Serum immunoglobulins (IgA, IgM, and IgG) play key roles in evaluating animal immune status and serve as the most important indicators of the humoral immune response in animals [19]. When bacteria or viruses infiltrate the body, they can bind to specific antigens, triggering the activation of humoral immunity. This immune response works to eliminate bacteria, viruses, and other pathogenic substances, playing crucial protective and defensive roles in the body [20]. Throughout the trial period, all the immunoglobulin indices were either better than or similar to those of the Control group, possibly because CMM-active components promote the development of immune cells and improve the immune response [21].

The biochemical indices in serum are influenced by the body’s metabolism and health status. Changes in these indicators reflect alterations in physiological processes [22]. The TP and ALB levels in serum are indicators of physiological health and homeostasis maintenance in animals [23]. Our results indicated that CMM did not significantly increase TP or ALB levels. In addition, the slight decrease in AST and ALT levels related to CMM suggests that CMM has no significant effect on liver function, as increased activity of these two enzymes generally indicates liver cell damage [24]. An increase in TG indicates elevated fat levels in blood; no notable changes in TG levels were observed in our study in response to CMM supplementation, but there was a slight decrease in TCH content that was not statistically significant. These findings indicate that CMM had no effect on blood lipid. Additionally, CMM did not significantly alter CREA or UREA levels in serum; CREA serves as an indicator of the glomerular filtration rate, and elevated levels of both of these markers indicate renal function impairment [25], implying that CMM did not induce renal damage in broilers. Thus, CMM has no discernible effect on the serum indices or health status of broilers.

In intensive poultry farming, several environmental factors and pathogens can weaken the immune system of poultry, affecting their intestinal health. Stimulation of the intestinal mucosa activates immune responses and the release of cytokines, such as the proinflammatory factors IL-1β, IL-6, IL-2, and TNF-α as well as the anti-inflammatory factor IL-10, all of which influence immune responses [26]. Our further analysis revealed that the TNF-α, IL-1β, IL-6 and IL-2 mRNA levels in the jejunum tissues of the CMM group were downregulated, whereas the levels of IL-10 and IFN-γ tended to increase. This finding is consistent with the results of Calik, who reported similar immune function regulation by vitamin E and selenium [27], indicating a suppression of IL-1β and TNF-α levels and an increase in IL-4 and IL-10 levels in the jejunum of broilers. The small intestine serves as a pivotal site for digestion and nutrient assimilation, and positive changes in VH and CD can positively affect the intestinal absorption of animals and indicate their status, [28]. In our study, the addition of low and high concentrations of CMM to the diet improved intestinal morphology, regulated VH and CD, and affected the intestinal digestion and absorption capacity of chickens to some extent. The effect of CMM addition on feed conversion may also be attributed to changes in intestinal morphology.

The gut microbial environment serves as a barrier against foreign pathogens, and modulation of the gut flora can impact metabolism and the immune response in animals [29]. Mao et al reported that fermented dandelion inhibited the proliferation of harmful bacteria and increased the abundance of beneficial bacteria, maintaining the flora balance and modulating immune capacity [30]. As a prospective feed supplement, the effects of CMM on intestinal flora need to be investigated. In our study, the addition of CMM increased the number of unique OTUs and α diversity in chickens. The findings indicated that incorporating a low concentration of CMM led to increased ACE and Chao 1 index values, indicating increased richness in the cecal flora. However, no level of CMM addition exerted any discernible effect on the Shannon and Simpson indices. This collective outcome suggests an overall improvement in the intestinal flora. Moreover, classification of the intestinal flora at the phylum level in broilers supplemented with CMM remained largely unchanged, maintaining the fundamental structure of intestinal flora. However, it may affect the abundance of microbes at lower taxonomic levels such as classes, orders, families, and genera. *Firmicutes* and *Bacteroidetes* are the main components of the intestinal flora, and it is generally speculated that an impaired intestinal barrier decreases the *Firmicutes*/*Bacteroidetes* ratio [31]. The incorporation of CMM increased the *Firmicutes*/*Bacteroides* ratio, which is similar to the results of Kong et al [32], in which the addition of glycerol monolaurate increased the *Firmicutes* abundance. *Deferribacteres* are bacteria that generate energy through obligate or facultative anaerobic metabolism and maintain iron balance in the intestine. We found that the abundance of *Deferribacteres* in the Low CMM group significantly increased at 14 d. Another study reported that the administration of probiotics resulted in an increase in the abundance of *Deferribacteres* [33]. Moreover, the Mid CMM group showed a reduced proportion of *Proteobacteria* at 14 and 28 d. Notably, *Proteobacteria* include various pathogenic bacteria, such as *E. coli*, which can secrete histamine [34], thereby inducing allergic or toxic reactions and promoting the occurrence of gastrointestinal disease [35]. This finding suggests that CMM can modulate gastrointestinal health.

At Days 14 and 28, CMM decreased the proportion of *Bacteroides* in all groups. Wu et al [36] observed a negative correlation between *Bacteroides* and immunoglobulins, suggesting that CMM may positively affect immune function. After 28 d of CMM feeding, the proportion of *Lachnospira* colonizing the gut increased. *Lachnospira* can produce nutrients such as butyrate, which provides energy to colon epithelial cells, maintaining immune homeostasis and promoting health [37]. *Synergistes*, a recently discovered genus, are widely distributed in anaerobic environments, including in soil and the intestinal tract [38]. Our study revealed that Low CMM concentrations increased the proportion of *Synergistes* bacteria at 14 d, whereas all CMM doses increased the proportion at 28 d, which is similar to findings that alfalfa flavones increased the abundance of intestinal *Synergistes* bacteria [39]. Furthermore, Li et al [40] found a negative correlation between syntrophic bacterial abundance and IL-1β, IL-6, and TNF-α levels, which is consistent with our findings suggesting that CMM may modulate inflammatory factors by altering intestinal microorganisms. During the 28-d feeding cycle, *Oscillospiraceae* were significantly enriched at all doses. This bacterial family is recognized for its capacity to synthesize short-chain fatty acids (SCFAs), particularly butyrate, through the degradation of different structural carbohydrates [41]. Moreover, *Oscillospiraceae* has been recognized as a promising candidate for developing probiotics [42]. In addition, *Megamonas*, classified under *Firmicutes*, has the capacity to ferment diverse carbohydrates, thereby promoting SCFA production in the intestine. We found that the proportion of *Megamonas* in the Low and Mid CMM groups increased at 14 and 28 d. Previous studies have shown that chicken samples fed phenylpyruvate have a relatively high proportion of *Megamonas* [43]. We also found that a Low CMM concentration increased the proportion of *Faecalibacterium*, which has been shown to be positively correlated with upregulation of IL-10 expression and can improve SCFA production and enhance intestinal immunity [44].

## CONCLUSION

In summary, the addition of 0.02% CMM to the base diet improved the growth performance, immunity parameters, intestinal morphology, and intestinal microbes of yellow-feathered broilers. This provides foundational support for the utilization of CMM as a poultry feed additive.

## Figures and Tables

**Figure 7 f7-ab-24-0791:**
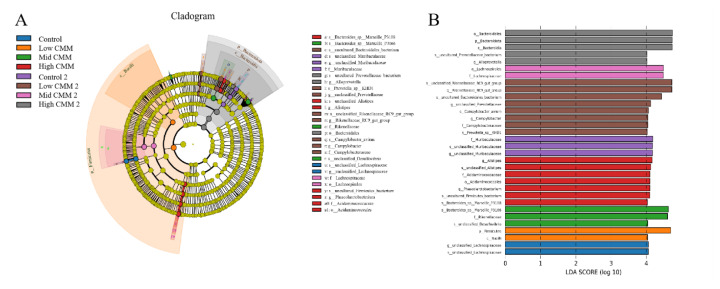
LEfSe analysis of the intestinal flora. (A) Cladogram. (B) LDA. CMM, *Cordyceps militaris* solid medium; LEfSe, linear discriminant analysis effect size; LDA, linear discriminant analysis.

**Table 1:** The ingredient composition and nutrient levels of the basal diets

Phases (%)	1–28 d	29–56 d
Ingredient^1)^	100	100
Corn	60.70	62.70
Soybean meal	30.50	28.00
Corn gluten meal	3.00	2.00
Soybean oil	1.50	3.00
Limestone	1.32	1.32
CaHPO4	1.33	1.33
Met	0.24	0.18
Middling	0.11	0.17
Premix	1.00	1.00
Nutrient^2)^		
ME/(MJ/kg)	12.29	12.76
CP	20.31	19.74
Lysine	1.01	1.1
Methionine	0.65	0.5
Calcium	0.87	0.88
Total phosphorus	0.66	0.57

1) The premix provided the following per kilogram of diet: 12,000 IU V_A_, 31,000 IU V_D_, 45 IU V_E_, 2.5 mg V_K_, 3 mg V_B1_, 5 mg V_B2_, 40 mg niacin, 15 mg pantothenic acid, 0.2 mg biotin, 1 mg folic acid, 20 μg V_B12_, 1,500 mg chloride, 80 mg Fe, 8 mg Cu, 100 mg Mn, 80 mg Zn, 0.7 mg I, and 0.2 mg Se.

2) The nutrient levels were calculated from data provided by the feed database in China.

ME, metabolizable energy; CP, crude protein.

**Table 2:** The nutrient levels in CMM

Nutrient	Contents (%)
Crude protein	18.2
Coarse fiber	4.0
Crude fat	2.8
Crude ash	2.7
Calcium	0.59
Phosphorus	0.12
Hydration	6.41

CMM, *Cordyceps militaris* solid medium.

**Table 3:** Sequence of primers for RT–PCR

Gene	Primer sequences (5′-3′)	Gene bank	Length (bp)
*β-actin*	Forward: TATGTGCAAGGCCGGTTTC Reverse: TGTCTTTCTGGCCCATACCAA	NM_205518.2	110
*IL-6*	Forward: CTCCTCGCCAATCTGAAGTC Reverse: CCTCACGGTCTTCTCCATAAAC	NM_204628.2	99
*IL-2*	Forward: CTTTGGCTGTATTTCGGTAGCA Reverse: CACTCCTGGGTCTCAGTTGGTG	NM_204153.2	168
*TNF-α*	Forward: GGACAGCCTATGCCAACAAG Reverse: GCGGTCATAGAACAGCACTAC	XM_046927265.1	81
*IL-1β*	Forward: CGACATCAACCAGAAGTGCTT Reverse: GTCCAGGCGGTAGAAGATGA	NM_204524.2	298
*IFN-γ*	Forward: GACAAGTCAAAGCCCCACAT Reverse: TCAAGTCGTTCATCGGGAGC	NM_205149.2	127
*IL-10*	Forward: CGCTGTCACCGCTTCTTCA Reverse: CGTCTCCTTGATCTGCTTGATG	NM_001004414.4	63

RT-PCR, real-time polymerase chain reaction; bp, base pair; IL, interleukin; TNF, tumor necrosis factor.

**Table 4:** The impact of CMM on growth performance of yellow-feather broilers

	Control	Low CMM	Mid CMM	High CMM	SEM	p-value
BW (g)						
1 d	759.31	755.79	763.38	755.23	3.34	0.077
14 d	1,282.74^b^	1,300.11^ab^	1,306.65^a^	1,297.31^ab^	6.65	0.010
28 d	2,106.18^b^	2,165.60^a^	2,161.73^a^	2,118.41^b^	12.28	0.000
ADG (g/d)						
1–14 d	37.39^b^	38.88^a^	38.81^a^	38.72^a^	0.41	0.003
15–28 d	58.82^b^	61.82^a^	61.08^a^	58.65^b^	0.72	0.000
1–28 d	48.10^b^	50.35^a^	49.94^a^	48.69^b^	0.43	0.000
ADFI (g/d)						
1–14 d	60.13^b^	60.86^b^	60.93^ab^	61.97^a^	0.41	0.001
15–28 d	159.55^a^	151.53^c^	153.89^b^	159.77^a^	0.46	0.000
1–28 d	109.84^a^	106.20^b^	107.41^c^	110.87^d^	0.33	0.000
FCR						
1–14 d	1.61	1.57	1.57	1.60	0.016	0.025
15–28 d	2.7^a^	2.45^b^	2.52^b^	2.73^a^	0.03	0.000
1–28 d	2.28^a^	2.11^b^	2.15^b^	2.28^a^	0.02	0.000

a–d A significant difference between the mean values is indicated by different lowercase letters in the same line (p<0.05), n = 8.

CMM, *Cordyceps militaris* solid medium; SEM, standard error of the mean; BW, body weight; ADG, average daily gain; ADFI, average daily feed intake; FCR, feed conversion ratio.

**Table 5:** Effects of CMM on immune organ indices

Organ	Day	Control	Low CMM	Mid CMM	High CMM	SEM	p-value
Spleen	D_14_	1.18^a^	1.27^a^	1.12^a^	1.12^a^	0.086	0.295
	D_28_	1.19^a^	1.37^a^	1.27^a^	1.19^a^	0.099	0.257
Thymus	D_14_	2.46^ab^	2.72^a^	2.50^ab^	2.32^b^	0.117	0.021
	D_28_	2.90^b^	3.29^a^	3.13^ab^	3.05^ab^	0.137	0.065
Bursa	D_14_	1.96^b^	2.28^a^	2.18^a^	1.95^b^	0.064	0.000
	D_28_	1.64^b^	1.93^a^	1.78^ab^	1.70^b^	0.068	0.002

a,b A significant difference between the mean values is indicated by different lowercase letters in the same line (p<0.05), n = 6.

CMM, *Cordyceps militaris* solid medium; SEM, standard error of the mean.

**Table 6:** Effects of CMM on serum biochemical indices

	Variable	Control	Low CMM	Mid CMM	High CMM	SEM	p-value
14 d	TP (g/L)	42.78	42.23	41.53	40.33	2.512	0.787
	ALB (g/L)	13.47	12.95	13.33	12.25	0.751	0.389
	AST (U/L)	250.67	250.50	247.17	248.00	9.252	0.974
	ALT (U/L)	4.47	4.30	3.77	3.95	0.475	0.456
	TG (mmol/L)	0.69	0.69	0.69	0.64	0.043	0.584
	TCH (mmol/L)	3.80	3.79	3.75	3.64	0.290	0.944
	UREA (mmol/L)	0.28	0.27	0.27	0.18	0.055	0.292
	CREA (μmol/L)	20.62	20.28	21.55	21.72	1.227	0.592
28 d	TP (g/L)	45.43	44.00	44.80	41.82	1.820	0.245
	ALB (g/L)	13.15	11.87	12.83	11.63	0.600	0.055
	AST (U/L)	230.67	205.83	226.17	208.33	11.094	0.086
	ALT (U/L)	4.28	4.15	3.30	3.97	0.449	0.164
	TG (mmol/L)	0.77	0.75	0.71	0.72	0.532	0.671
	TCH (mmol/L)	3.18	2.95	3.07	2.90	0.228	0.640
	UREA (mmol/L)	0.50	0.38	0.45	0.48	0.059	0.243
	CREA (μmol/L)	25.53	25.22	24.20	21.98	1.786	0.218

n = 6.

TP, total protein; ALB, albumin; AST, aminotransferase; TG, triglyceride; TCH, total cholesterol; UREA, urea; CREA, creatinine.
